# SARS-CoV-2 Vaccination in India: Considerations of Hesitancy and Bioethics in Global Health

**DOI:** 10.5334/aogh.3530

**Published:** 2021-12-10

**Authors:** Mohammad Abdullah Sarkar, Ahmad Ozair, Kaushal Kishor Singh, Nishanth R Subash, Mainak Bardhan, Yashita Khulbe

**Affiliations:** 1 Department of Psychology, Aligarh Muslim University, Aligarh, Uttar Pradesh, India; 2 Faculty of Medicine, King George’s Medical University, Lucknow, Uttar Pradesh, India; 3 Department of Neurology, National Institute of Mental Health and Neuro-Sciences, Bengaluru, Karnataka, India; 4 Division of Bacteriology, National Institute of Cholera And Enteric Diseases, Kolkata, West Bengal, India

## Abstract

**Introduction:**

Worldwide mass vaccination against SARS-CoV-2, while having been the most critical action in combating further waves of COVID-19, was initially fraught with multiple infrastructural and socio-cultural challenges. Vaccine hesitancy, a phenomenon of doubt over the vaccines’ claimed efficacy and/or safety amidst access to vaccination, emerged as a major challenge for global health, despite approval and regular post-marketing surveillance by major regulatory bodies.

**Methods:**

We reviewed the literature related to vaccine hesitancy in India published until November 14, 2021 using relevant keywords in various databases and examined it from a bioethical perspective.

**Results:**

Factors driving hesitancy either intensified skepticism towards vaccination in general or exacerbated reluctance towards specific vaccines. In India, hesitancy towards indigenously developed vaccines was aggravated by the lack of peer-reviewed phase III trial data before the start of vaccination, lack of public transparency of regulatory bodies, and presence of public perception of inappropriately expedited processes. This perspective piece discusses the state of mass immunization in India as a case of how vaccination and its hesitancy thereof gave rise to unique bioethical challenges in global health. In early 2021, vaccination in India was subject to difficulties in adhering to the principles of equity and justice, while a compromise of the principles of informed consent, beneficence, and non-maleficence also perhaps did occur.

**Conclusions:**

Post-pandemic debriefing on the subversion of bioethical principles will be needed, and an appropriate response may be required to rebuild and enhance the public faith in future mass vaccination movements.

## Introduction

The development of safe and effective vaccines against SARS-CoV-2 has been a key juncture in the global response to the pandemic. As of November 14, 2021, the World Health Organization has approved eight vaccines [1].

While worldwide mass vaccination has become the most critical action in combating further COVID-19 waves, it has been fraught with multiple infrastructural and socio-cultural challenges. These difficulties have been particularly accentuated with regards to low- and middle-income countries (LMICs) with far lower proportions of their populations having been vaccinated compared to high-income countries (HICs). Heavily populous LMICs have especially struggled to procure and administer vaccines on a timely basis.

While manufacturers and policymakers have worked together to alleviate the infrastructural challenges behind vaccine shortages, vaccine hesitancy has emerged as a major impediment [2]. This phenomenon of doubt over the vaccines’ reported efficacy and/or safety amidst access to vaccination, despite approval and regular post-marketing surveillance by major regulatory bodies, was a complex byproduct of multiple factors. These factors may have either intensified hesitancy towards all vaccines in general or may have exacerbated reluctance towards specific vaccines. The former group of factors include region-specific socio-cultural factors, low levels of health literacy, spread of misinformation, religious beliefs, and history of non-utilization of national health services pre-pandemic, amongst others. The latter include, but are not limited to, rare adverse events temporally related with the vaccine, non-transparency from regulatory authorities, misinformation regarding specific vaccines, and so on [2].

This viewpoint uses the case scenario of India to discuss vaccine hesitancy and its implications in order to highlight bioethical issues in global health systems, including inequitable vaccination practices, inappropriate informed consent, and weighing beneficence and non-maleficence, amidst a state of uncertainty.

## State of SARS-CoV-2 Vaccination in India

In the second-most populous country in the world, with nearly 1.3 billion individuals, mass vaccination initially had a slow start but then gradually gained speed. Notably, by the end of July 2021, coinciding with the end of the last peak of cases in India, India had suffered 420,000 officially reported deaths, while merely 6.8% of the population had been fully vaccinated [3]. This is even though Serum Institute of India, a company based in Pune, India, has historically been the largest manufacturer of vaccines in the world. The one billionth dose of COVID-19 vaccines was administered in October; however, only 31% of adults had been fully vaccinated [4].

Currently, six different vaccines are being administered across India, with the backbone of national vaccination programme being Covaxin and Covishield. Covaxin (BBV152), developed and manufactured by Bharat Biotech International, is India’s fully indigenous vaccine. Covishield is an iteration of the Oxford/AstraZeneca vaccine (AZD1222) being manufactured by the Serum Institute of India. In addition, Sputnik V and the Moderna COVID-19 vaccine (mRNA-1273) have also available since the end of July 2021. The Janssen vaccine (JNJ-78436735), along with India’s locally developed ZyCoV-D, are the latest additions to India’s vaccination program, but have very low contribution to the total vaccine doses administered in the country. Notably, Covaxin and Covishield are amongst the major pillars of COVAX, which is the World Health Organization’s (WHO) vaccine-sharing initiative [5].

## Vaccine Hesitancy in India

Vaccine hesitancy had been widespread in India for a significant part of 2021, despite multidisciplinary efforts at tackling it. Public surveys portrayed a high rate of vaccine hesitancy initially among the general population while state reports further corroborated such statements [2,6]. This hesitancy was not merely been limited to the general populace alone. A publication reported that nearly 55% of healthcare workers had admitted when surveyed in early 2021 that they were reluctant to take the vaccine or had not decided upon it [7]. This hesitancy persisted despite the major part of the Indian vaccination system being dependent on the two indigenously manufactured vaccines.

Concerns regarding safety and efficacy over social media were found to be the prime reasons behind vaccine hesitancy [8]. Additionally, multiple incidents highlighting the deaths of vaccine recipients exacerbated the public distrust. Relatives of the people who died or were hospitalized after vaccination in India claimed that the officials did not reply to any queries being posited about the vaccine being a possible contributing factor towards these deaths [9]. Misinformation, especially through social media, and religious beliefs further contributed to vaccine hesitancy.

Policymakers and health officials attempted to tackle hesitancy through multipronged efforts. A “time-bound investigation” was ordered into deaths that occurred soon after vaccination, while each mobile phone call in the country was automatically preceded by a national programmed message from the Government of India endorsing the safety and efficacy of vaccines [10].

## Vaccine Hesitancy in Other South Asian Countries

High levels of vaccine hesitancy were reported in other South Asian countries as well, a significant blow to the global pandemic response and concern for the emergence of future variants secondary to SARS-CoV-2 replication in high-density populations. These nations were also impacted given that it was India supplying its indigenous vaccines to many of these countries. Therefore, the additional contribution to vaccine hesitancy associated with the indigenous nature of Covaxin and Covishield transcended national borders.

A similar struggle was noted in Pakistan in its vaccination drive, with the rate of inoculation being 0.2 vaccinations per 100 people as of March 2021. The low vaccination rate was attributed to misinformation and general distrust in the world’s fifth-most populous country [11,12].

In Bangladesh, less than half the citizens were willing to get vaccinated even if the inoculation was free of cost. Notably, residents of slums and rural areas were the most hesitant towards vaccines [13]. Upon receiving Covishield doses from India, the Bangladesh health minister had to publicly request the citizens to “not pay heed to rumors and take the vaccine” [14]. These concerns were exacerbated by the reports of fake vaccines being manufactured, smuggled, and inoculated in Asia which may have further intensified people’s hesitancy. Interpol had already warned of more fake vaccines in the future [15].

Nepal saw its vaccination program also come to a halt after India stopped sending its vaccines as the latter dealt with rising COVID-19 cases coupled with acute vaccine shortage [16]. Healthcare and sanitation workers in Nepal were also hesitant against getting vaccinated due to poor outreach and vaccination strategy [17,18].

In Afghanistan, merely 4% of the population was vaccinated by early July 2021, whilst the country was facing a third wave of the pandemic [19]. Vaccination here occurred amidst violence and inadequate health facilities, aimed at just 20% of the population [20]. For instance, the Taliban allowed support from COVAX officials in Afghanistan as long as they did not visit Afghani residents [21].

Sri Lanka also found its residents hesitant in early 2021 with an online survey reporting that most of the population was still concerned about the nature and efficacy of the vaccines [22]. The minister for the prevention of COVID-19 in Sri Lanka highlighted how deaths after inoculation were erroneously linked to vaccines and were caused by other complications [23].

## Vaccinating Before Availability of Peer-Reviewed Phase 3 Trial Data

To understand the bioethical concerns behind vaccination and, to some extent, vaccine hesitancy in India, it is essential to review the development of vaccines that are available here. While the country attempted to manufacture an adequate number of doses of indigenous vaccines, however, their approval and post-marketing surveillance procedures were less than ideal.

Covaxin has been developed and is being manufactured by Bharat Biotech in collaboration with the Indian Council of Medical Research (ICMR) and the National Institute of Virology, Pune. It is based upon whole-virion Vero cell-derived platform technology, employing inactivated viruses [24]. Covishield, the vaccine developed under Oxford-AstraZeneca collaboration, is being manufactured by Serum Institute of India. It utilizes a viral vector pattern, whereby an adenovirus is modified to mimic the protein spikes present on top of SARS-CoV-2 [25]. The Sputnik V is also an Adenoviral vector-based vaccine. It was developed by Gamaleya National Research Center, Russia and is being produced by Serum Institute of India and Dr. Reddy’s Laboratories [26]. Unlike Covaxin and Covishield, Sputnik V’s second dose has a different vector than the first to enhance efficacy [27]. The Janssen vaccine is also a viral vector vaccine developed by Janssen, Johnson & Johnson’s pharmaceutical wing [1]. India’s indigenously developed ZyCov-D vaccine is the world’s first DNA vaccine against COVID-19 and has been tested on young individuals. The vaccine, developed by Cadila Healthcare, uses plasmids to deliver a needle-free jab and requires three doses [1]. All vaccines being administered in India, with the exception of Sputnik V and ZyCov-D, have been approved by the WHO as of November 14, 2021, with the latest addition being Covaxin in November itself [1].

The Indian government administered far more doses of Covishield than Covaxin with the latter making up just 11% of the total doses administered, as reported by the CoWIN dashboard of the Government of India. Meanwhile, the Sputnik V has been administered around 5 million times in India as of November 6, 2021 [28]. This preference of Covishield was also seen in the increased order volume placed by the government for it [29].

An initial preference for Covishield in the Indian populace was rooted in Covishield’s due completion of trials coupled with apprehensions regarding the efficacy and safety of Covaxin [29,30]. Meanwhile, the Indian government did attempt to reduce the hesitancy that surrounded Covaxin’s uptake in the population [31]. The hesitancy particularly around Covaxin may be mainly attributed to lack of peer-reviewed phase 3 trial data, which is also why it remained unapproved by the WHO for the major part of 2021 [31]. Notably, it was only after the release of this phase 3 trial data by Bharat Biotech that Covaxin got WHO’s approval on November 4, 2021 for emergency use [32]. As for Sputnik V, the rollout continued to be slow in the absence of an adequate number of doses [33]. Unfortunately, Sputnik vaccine continues to remain unapproved by the WHO till date (14^th^ November, 2021) [34]. Data on vaccine-wise trends has not been made available on the CoWIN dashboard for Janssen and ZyCov-D.

The commercial production of Covishield was approved after AstraZeneca had already published the results of an interim analysis of its phase 3 clinical trials [35,36]. Meanwhile, India resorted to using Covaxin on the population before the publication of even peer-reviewed phase 2 trial data. Mass inoculation using Covaxin had started in January 2021 after approval from India’s Central Drugs and Standards Committee (CDSCO), at a time when only a preprint of its phase 2 trial data was available [36,37]. The All-India People’s Science Network and the All-India Drug Action Network, which are both networks of several scientific and non-government organizations, severely criticized this decision. However, the CDSCO moved ahead. By June 2021, despite thousands of doses administered, Covaxin’s manufacturers had released only an unpublished interim analysis of phase 3 data [38], Covaxin’s trials did not conclude till July [39]. By the end of July 2021, Covaxin, along with Sputnik V, lacked WHO’s approval [27]. Meanwhile, Moderna’s vaccine, which was backed up by high-quality peer-reviewed phase 3 trial data, was approved in India but very few doses had been administered [40].

The state of approval of indigenous vaccines in India was in stark contrast with the regulatory processes that the high-income countries (HICs) utilized. The vaccines being used by the United States and the United Kingdom had their phase 3 trial data duly published and peer-reviewed before their use outside of trial purposes [40,41,42]. The careful administration of vaccines and the following of due protocols likely had impacted the way citizens perceived the efficacy and safety of vaccination in HICs.

## Ethical Issues in Informed Consent of Vaccination

Informed consent is the practice of ensuring that subjects, both within a trial and outside, understand the need, risks, benefits, and alternatives before an intervention is carried [43]. Essential elements of informed consent include proper disclosure of information to the subjects, the subject’s comprehension of the information provided, and the voluntary nature of the decision. For interventions with a significant degree of risk, injury compensation is usually provided.

Severe ethical lapses, including inadequacy in consent forms and the lack of documentation or compensation, plagued the Covaxin rollout [44,45]. Several independent reports suggest that appropriate implementation of informed consent protocols, both within and outside trials, was lacking [43,44,45].

As commercial production was approved, a series of guidelines were announced for overseeing the administration of Covaxin and Covishield. However, adherence to these guidelines was less than adequate [46]. The administration of Covishield required no consent form but was needed for Covaxin up till March 2021, when the guidelines were revised following the interim analysis (but not publication) of Covaxin’s phase 3 trial data [47]. In contrast, written consent was not required in the US as the vaccine authorization came after the publication of phase 3 trial data [48,49].

In an intervention with emerging evidence, it is essential that prospective participants receive all necessary information to understand what they are volunteering for and the risks of participating [50]. Yet, in the case of Covaxin, many subjects before inoculation were not informed that evidence regarding the safety and efficacy of the vaccine was not yet available. Recipients in some areas claimed they were not counseled about the risks of the study altogether [51,52]. This was further exacerbated by the reports of vaccine-induced thrombotic thrombocytopenia (VITT), especially cerebral venous sinus thrombosis (CVST), in those inoculated with the AstraZeneca vaccine [53]. One co-convener of All-India Drug Action Network described on July 28, 2021, how VITT cases in India were not being properly investigated [54]. Such trial protocol violations, if correct, were concerning and raised questions about the standards of research ethics in India, given these were critical trials that should have faced higher scrutiny.

It is a cardinal principle of bioethics to have trial participants be aware that they are indeed participating in a trial, with rare exceptions such as emergency research in a mortal situation and therapeutic privilege of withholding information for the welfare of the patient [55]. However, many Covaxin-inoculated subjects were noted to report that they did not know about being enrolled in a trial [56]. Such non-informed participation carries grave considerations, particularly when looked through lens of historical injustices done in biomedical research to marginalized populations. Notably, the subjects in the prior report were from a slum in Bhopal, a city in Central India [56]. Recruiting participants from low-income areas without obtaining informed consent appropriately, if true, was a serious lapse in ethics.

It is important to emphasize that while Covaxin did ultimately gain the approval of WHO, the trial conduct does not justify the trial outcomes. The manner of Covaxin administration remains concerning from a bioethical perspective, which stresses upon the means used to achieve an outcome rather than just focusing on the outcome alone. Therefore, coincidentally positive outcomes do not justify ethically challenged processes in global health as these create dangerous precedence for the future [57].

## Considering Beneficence and Non-Maleficence Amidst Uncertainty

The principle of non-maleficence states that one must not intentionally harm subjects [58]. The principle of beneficence, which states that one ought to work for others’ benefit, is often in conflict with the idea of non-maleficence, given that medical interventions usually carry both risks and benefits. Beneficence usually takes precedence over non-maleficence when a socially valuable outcome is involved.

Given that the failure rate of drugs and devices in phase 3 trials is known to be extremely high, trials are essential to assess both the safety and efficacy of interventions before their approval or emergency use authorization [59]. Such practices maximize benefit while minimizing number of subjects that may be harmed through non-efficacious or harmful interventions. Unfortunately, this was not followed when Covaxin received approval from CDSCO despite lacking peer-reviewed data of even the phase 2 trials. This was a major lapse in global health ethics, for which post-hoc administrative transparency would be helpful in building trust in public health systems.

Meanwhile, Covishield was approved based on trial data from the USA, Brazil, and South Africa, without completing adequate bridge trials, a concern that was raised by a few experts [60]. Bridge trials are conducted as per existing clinical practices on interventions that have undergone phase 3 trials in other environments and conditions to ensure that they are safe for the local population in particular. Some health experts in India did advocate for waiver of bridge trials to facilitate better inoculation rates, while others disagreed [61,62].

Thus, the importance of standard operating procedures (SOPs) remained upheld for HICs but was disregarded, in favor of urgency, for LMICs. SOPs provide safeguard against damages that would impact the healthcare and economic system of a country; LMICs, by their very definition, already struggle with both the sectors. Thus, bypassing SOPs is a more serious concern when associated with LMICs.

While the efficacy of SARS-CoV-2 vaccines, in general, has been excellent, evidence continued to emerge gradually throughout 2021 regarding their rare adverse effects, particularly regarding vaccine-induced thrombotic thrombocytopenia and vaccine-associated myocarditis [53]. Ideally, large public health interventions should have been carried out with due assurance of their safety through published, peer-reviewed phase 3 trial data, where the number of participants are much higher than previous phases and the possibility of detecting rarer adverse events is greater. However, this was carried out sub-optimally in the case of indigenous vaccines. Meanwhile, the insufficient post-marketing surveillance (Phase 4 trials) of vaccines in India and the lack of timely publication of the national data of such events perhaps led to an incorrect perception of risks in those getting vaccinated. This also imbalanced the scale of weighing beneficence versus non-maleficence.

The Indian Medical Association (IMA), the largest association of physicians in India, highlighted that emergency approval for vaccines in India had more disadvantages than advantages and suggested that clinical trials should have included volunteers from diverse backgrounds to solidify the vaccine’s safety record. Early in 2021, the IMA announced their reservation against the proclamation of a vaccine being completely safe based on unpublished interim analysis alone [62]. Such reservations from a professional society did raise concerns; however, little was done to formally reach out to alleviate them.

## Need for Justice and Equity in Global Vaccination Practices

The principles of justice and equity dictate that the projected risk and benefits should be ideally shared proportionately across represented groups [63]. However, the inequitable manner of worldwide vaccine distribution posited a threat to these principles. Questions must be raised over vaccine nationalism and vaccine apartheid, the intent of nations to acquire a vaccine for themselves with little or no regard for other countries. As highlighted by the WHO, such practices are derogatory to these principles and can potentially lead to an impending “catastrophic moral failure” [64].

Throughout 2021, countries with the high levels of income per capita and healthcare placed the most orders for the WHO-approved vaccines. Meanwhile, only 0.2% of global vaccine exports were directed towards lower-income countries, creating a concerning health disparity [65]. Over 10 billion doses of the COVID-19 vaccines have been ordered, of which half have been ordered by 32 countries alone, equaling merely 13% of the global population [66].

Such orders resulted in a skewed distribution from early 2021 alone, when India lacked the necessary two doses per person while Canada had nine doses per person. Similarly, over 130 countries awaited the availability of the appropriate number of first doses [67]. While India attempted to bridge this gap by pledging to export vaccines, it had to halt exports given the rising COVID-19 cases in the middle of 2021 [68].

Lack of equitable vaccine availability in global health ultimately harmed all countries. Allowing for COVID-19 spread in non-vaccinated populations in LMICs allowed SARS-CoV-2 to replicate and, thereby, generate novel, more contagious variants, resulting in loss to societies worldwide [69]. The selfishness of developed nations thus harmed not just the poorer ones, but the high-income countries themselves as well, because containing SARS-CoV-2 variants within a single country’s borders proved to be extremely difficult. Post-pandemic debriefing should discuss how the phenomena of vaccine apartheid and vaccine nationalism thus greatly harmed global health.

## Conclusions

**Figure 1:**
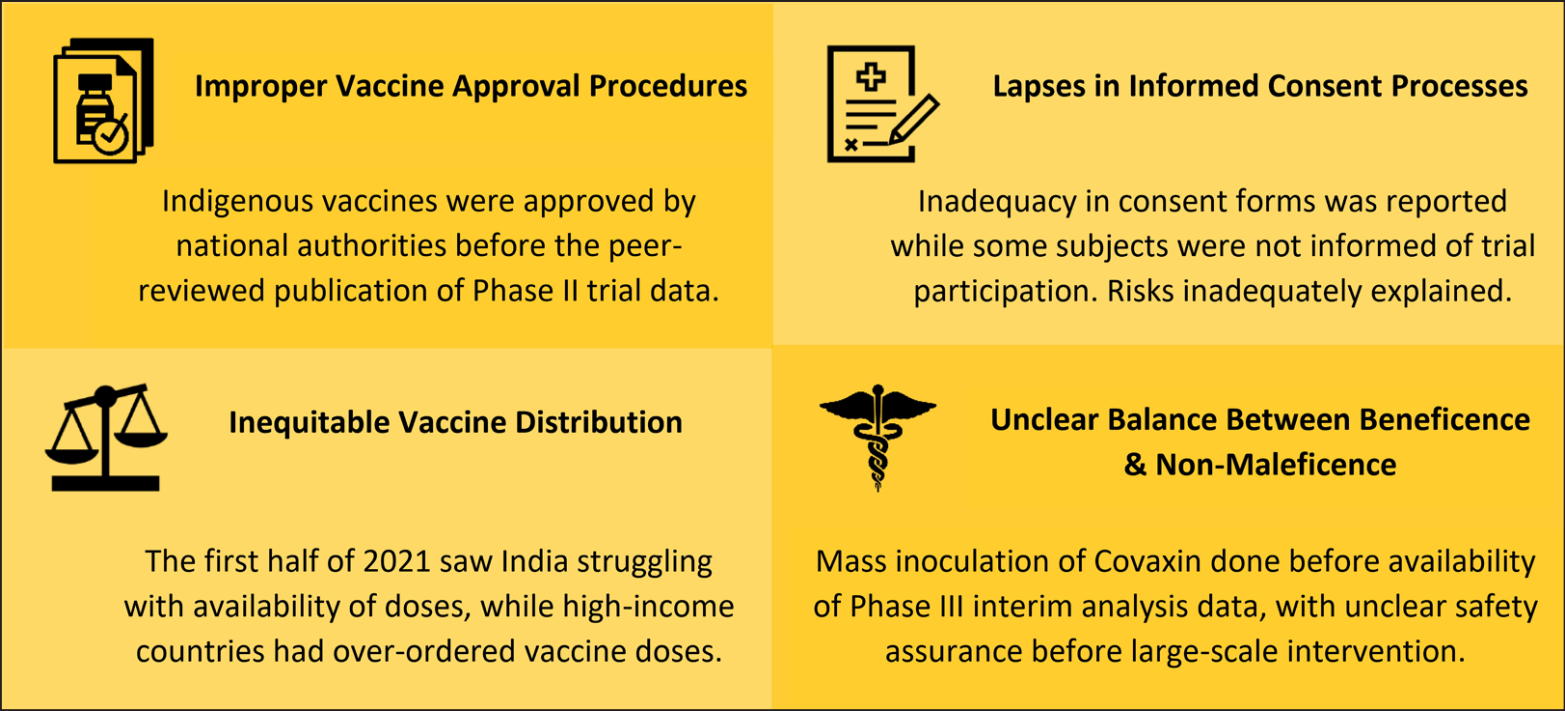
Summary of bioethical concerns behind the mass vaccination programme against SARS-CoV-2 in India.

Vaccination and its hesitancy thereof gave rise to unique bioethical challenges in global health. Low- to middle-income countries (LMICs) like India initially faced a significant barrier to mass immunization in vaccine hesitancy (***Figure 1***). This hesitancy, while multifactorial in origin, was particularly exacerbated in India given the indigenous development of its vaccines, the lack of peer-reviewed phase 3 trial data, and the deficits in public transparency of regulatory bodies. Vaccination here had been subject to difficulties in adhering to the principles of equity and justice, while a compromise of the principles of informed consent, beneficence, and non-maleficence had also perhaps occurred. Post-pandemic debriefing on the subversion of bioethical principles will be needed to understand and appropriate response required to regain the public’s trust in future mass vaccination movements.
